# Locoregional Therapy Approaches for Hepatocellular Carcinoma: Recent Advances and Management Strategies

**DOI:** 10.3390/cancers12071914

**Published:** 2020-07-15

**Authors:** Mina S. Makary, Umang Khandpur, Jordan M. Cloyd, Khalid Mumtaz, Joshua D. Dowell

**Affiliations:** 1 Division of Vascular and Interventional Radiology, Department of Radiology, The Ohio State University Wexner Medical Center, Columbus, OH 43210, USA; umang.khandpur@osumc.edu; 2 Division of Surgical Oncology, Department of Surgery, The Ohio State University Wexner Medical Center, Columbus, OH 43210, USA; jordan.cloyd@osumc.edu; 3 Division of Hepatology and Gastroenterology, Department of Internal Medicine, The Ohio State University Wexner Medical Center, Columbus, OH 43210, USA; khalid.mumtaz@osumc.edu; 4 Northwest Radiology, Indianapolis, IN 46290, USA; josh.dowell@gmail.com

**Keywords:** hepatocellular carcinoma, transarterial embolization, chemoembolization, radioembolization, ablation, immunotherapy, TAE, TACE, TARE, SIRT

## Abstract

Hepatocellular carcinoma (HCC) is the most common primary liver malignancy and third leading cause of cancer-related mortality worldwide. While surgical resection and transplantation are the standard first-line treatments for early-stage HCC, most patients do not fulfill criteria for surgery. Fortunately, catheter-directed and percutaneous locoregional approaches have evolved as major treatment modalities for unresectable HCC. Improved outcomes have been achieved with novel techniques which can be employed for diverse applications ranging from curative-intent for small localized tumors, to downstaging or bridging to resection and transplantation for early and intermediate disease, and locoregional control and palliation for advanced disease. This review explores recent advances in liver-directed techniques for HCC including bland transarterial embolization, chemoembolization, radioembolization, and ablative therapies, with a focus on patient selection, procedural technique, periprocedural management, and outcomes.

## 1. Introduction

Hepatocellular carcinoma (HCC) is the most common primary liver malignancy [1]. The prognosis depends on a multitude of clinical, laboratory, and radiologic parameters, but the overall 5-year survival rate for liver cancer remains below 20% [2,3,4]. Traditional management options for patients with HCC include surgical resection and orthotopic liver transplantation (OLT) [4]. However, in a recent comparative study of more than 8000 HCC cases worldwide, less than 10% of patients fulfilled preoperative criteria for resection [5]. For patients who are not ideal surgical candidates, novel liver-directed strategies are being utilized to treat appropriately selected patients, and in some cases achieve curative effect. Other goals of locoregional approaches include tumor cytoreduction for downstaging or “bridging” to maintain eligibility for transplantation, hypertrophy of hepatic tissue to increase liver function for future major resection, and palliation [6]. Over the past two decades, management approaches that increase overall survival and reduce adverse effects for a wide range of patients have increased with the incorporation of new image-guided techniques and enhanced targeted pharmaco- and radiotherapeutics [7,8]. This review discusses the recent advances in locoregional therapy for HCC including transarterial embolization (TAE), transarterial chemoembolization (TACE), transarterial radioembolization (TARE), and ablative therapies (Table 1).

## 2. Transarterial Embolization

### 2.1. Procedure

Intraarterial therapies are centered on the principle that hepatocellular tumors mainly recruit hepatic artery branches for growth whereas normal liver parenchyma receives dual blood flow primarily via the portal vein [7,9,10]. The goal of transarterial embolization (TAE) is to restrict hepatic artery blood flow to vessels supplying a tumor. This causes ischemia-induced cellular membrane disruption and oncosis resulting in ischemic cell death [11]. Both particulate and liquid materials can be used as embolic agents [12]. TAE is also known as “bland” embolization because the particles themselves are not equipped with additional functions such as chemotherapy or radiation.

During the procedure, identification of key hepatic artery branches supplying the tumor is crucial to maximize treatment effectiveness and avoid collateral ischemia to adjacent liver parenchyma. The targeted tumoral arterial supply is then treated with an embolic agent, most commonly microparticles ranging from 40 to 120 μm in size [13]. Depending on the disease distribution within the liver, the treatment approach can vary including lobar treatment for multifocal disease or targeted segmental treatment for unifocal disease [14]. The procedure is commonly performed in the inpatient setting under moderate sedation, although some patients may require general anesthesia [15,16,17].

The most common associated risk is that of postembolization syndrome (PES), the severity and duration of which might be correlated with the degree of healthy tissue ischemia and underlying liver function [18,19]. Additional risks include hepatic decompensation, renal injury, biliary injury, infection (abscess), and rarely pulmonary embolization via undetected arteriovenous shunts within the tumor and/or small embolic particle size [20,21,22]. Lastly, non-target embolization of extrahepatic vascular supply such as the cystic artery to the gallbladder is another risk [23]. Meticulous technique including the use of novel intraprocedural technologies such as cone-beam CT is utilized to ensure complete tumoral coverage while avoiding non-target embolization [24].

### 2.2. Periprocedural Management

Prior to the procedure, patients may be administered prophylactic antibiotics for coverage of gram-negative enteric microbes. Watchmaker et al. evaluated the need for infection prophylaxis before hepatic embolization and found that sterile technique of the procedure itself is enough to perform the procedure safely in patients with an intact sphincter of Oddi [25]; however, in patients with previous biliary or bowel interventions leading to altered sphincter function, the risk of postprocedural infection is significantly higher [26]. Thus, antibiotic administration and bowel preparation may be beneficial in these patients. For example, Khan et al. found that 400 mg of oral moxifloxacin given 3 days before and 17 days after hepatic transarterial therapy in patients at high risk for hepatic abscess formation was effective in preventing this complication [27]. Another protocol involves levofloxacin and metronidazole daily for 2 days preceding the procedure and continued for 2 weeks after combined with a bowel regimen of neomycin plus erythromycin at 1, 2 and 11 pm of the day before embolization [28]. Other preprocedural considerations include hydration status, antiemesis, antihistamines, and steroids. Some institutions use dexamethasone and hydrocortisone.

After the procedure, adequate hydration, pain and nausea control, and stable hepatic function tests are key criteria for discharge. PES is the most common complication of embolotherapy and presents with right upper quadrant pain, nausea, fatigue, fever, hypertransaminasemia, and hyperbilirubinemia [18,19,29]. It usually occurs within 72 h of the procedure but is self-limiting in most cases, and completely resolves in 7 to 10 days [29]. Debate exists regarding routine administration of postprocedural antibiotics, and this should be case-dependent until more robust data are available [30]. Specifically, patients with any history of biliary abnormality, bilio-enteric intervention, or dysfunctional sphincter of Oddi should likely continue antibiotics for 2 weeks [27]. Appropriate periprocedural anticoagulation management guidelines should be observed [31,32]. Follow-up imaging and laboratory investigations are conducted 4–6 weeks later and then every 3–6 months thereafter to evaluate treatment success as well as monitor disease progression [10,33]. CT or MRI can be used to confirm tumor necrosis [14].

### 2.3. Patient Selection

**Figure 1:**
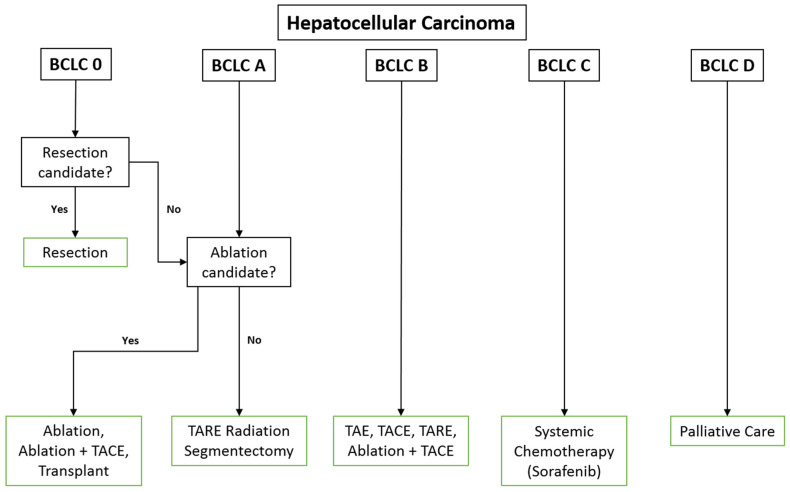
Treatment options for hepatocellular carcinoma stratified by BCLC class. Note that any locoregional approach can be used to maintain or downstage to transplant eligibility. Contraindications to resection include significant portal hypertension, hyperbilirubinemia, multiple nodules, and comorbidities. Contraindications to ablation include lesion > 5 cm, > 4 nodules, and anatomic infeasibility.

Generally, TAE is reserved for non-surgical candidates with liver-dominant disease. Patient selection for all locoregional therapies including TAE involves clinical and serologic evaluation of the patient including functional status, liver function tests, and clinical indices such as the ALBI (Albumin-Bilirubin), CP (Child-Pugh), MELD (Model for End-stage Liver Disease), and ECOG (Eastern Cooperative Oncology Group) performance status scores for patient stratification and assessment [34,35,36]. In addition to its role in the diagnosis of HCC, preprocedural imaging is paramount for evaluation of the vascular anatomy, access site patency, and ensuring patency of the portal vein [37]. Studies have demonstrated that patients in class B of the Barcelona Clinic Liver Cancer staging classification system (BCLC) derive the most benefit from this procedure followed by BCLC class C [10,38]. Patients in BCLC class A may undergo TAE to maintain eligibility for transplantation per the Milan and UCSF criteria [38,39]. The contraindications for TAE include decompensated cirrhosis (Child-Pugh B8 or higher), significantly reduced portal venous flow, creatinine clearance < 30 mL/min, high tumor burden, severe comorbidities, untreated esophageal varices, and elevated liver function markers [40]. Figure 1 shows possible treatment strategies stratified by BCLC class.

### 2.4. Prognostic Factors and Outcomes

Various clinical staging systems are currently in use for predicting overall survival, progression-free survival, and adverse events after intra-arterial therapy for HCC. Examples include the Okuda system, Cancer of the Liver Italian Program (CLIP) score, Hong Kong Liver Cancer (HKLC) staging system, and the Barcelona Clinic Liver Cancer (BCLC) classification scheme [41,42]. Among specific prognosticators, tumor burden, hepatic reserve, extrahepatic spread, and performance status are most strongly associated with overall survival in HCC [41,43,44,45]. The gold standard for assessing response to treatment is the 2010 modified Response Evaluation Criteria in Solid Tumors (mRECIST) [46].

Although outcomes comparing locoregional therapy to best supportive care are better characterized for TACE than for bland embolization, embolotherapy as a whole confers significant survival benefit compared to best supportive care [10,47]. Tsochatzis et al. [43] published results from a meta-analysis of six randomized controlled trials comparing TAE with TACE, and none of them revealed significant differences in overall survival [43,48]. Lee et al. [49] summarized evidence from three studies revealing no significant differences in 3-year survival rates, adverse events, or RECIST responses [49,50,51,52]. Interestingly, Kluger et al. [50] found that TAE patients were significantly less likely to require retreatment before transplantation than TACE patients. Finally, a 2009 multicenter RCT that compared drug-eluting bead transarterial chemoembolization (DEB-TACE) to TAE found significant improvement in time to progression in the DEB-TACE group, but no change in overall survival [53]. Since induced ischemia from embolotherapy could be the dominant contributor to tumor cell death and bland embolization does spare the cost of chemotherapy and its unfavorable toxicity profile, [54,55] TAE should continue to be offered to appropriately selected patients.

## 3. Transarterial Chemoembolization

### 3.1. Procedure and Periprocedural Management

Similar to bland embolization, transarterial chemoembolization (TACE) involves occlusion of tumor feeding vessels. In contrast to TAE, TACE permits the delivery of targeted chemotherapy with the embolic therapy. In the conventional approach (c-TACE), a lipiodolized chemotherapeutic agent is administered into a feeding artery followed by administration of an embolic agent. This theoretically allows for (1) increased pharmacologic concentration and (2) increased effect duration due to decreased washout [7,10]. However, there is considerable variation in technique and drug mixture across institutions, and pharmacokinetic analysis revealed that plasma concentration after c-TACE may approximate systemic chemotherapy drug levels [56]. A newer approach using drug-eluting beads (DEB-TACE) provides better standardization and arguably less hepatotoxicity [57,58]. In DEB-TACE, drug-infused microspheres release chemotherapy in a sustained fashion and serve an embolic role are injected. Doxorubicin is the most widely cited chemotherapeutic agent, but others use a solution adding mitomycin C and cisplatin in c-TACE [59].

Periprocedural evaluation and management is identical to that for TAE (see Section 2.2.). Antimicrobial prophylaxis is recommended as is periprocedural clinical stabilization and laboratory monitoring. PES is common after TACE occurring in up to 80% of patients [60]. Pharmacological treatments include intra-arterial lidocaine, steroids, 5-HT3R antagonists, and antibiotics. Antibiotics seem to be of little clinical utility in managing fever [61], but intra-arterial lidocaine and/or dexamethasone have improved analgesic requirements and hospital length of stay [62,63].

### 3.2. Patient Selection

The most appropriate candidate for TACE is one with intermediate-stage HCC (BCLC class B, Child-Pugh B or better) without portal vein thrombosis or extrahepatic spread who is ineligible for surgical resection or transplantation [10,64,65]. Numerous studies confirm that TACE can significantly impact survival if patients are selected on the aforementioned factors. For example, Burrel et al. [66] demonstrated a median survival up to 47.7 months after TACE for BCLC B patients with preserved liver function (no higher than Child-Pugh B7), no vascular invasion, extrahepatic spread, nor significant functional impairment [7,66].

BCLC A (early-stage) or BCLC 0 (very early-stage) patients with a solitary nodule and minimal to no underlying liver disease should undergo surgical resection which boasts a favorable prognosis [7]. However, TACE may be indicated in these patients especially if they are ineligible for surgery/ablation or require a “bridge” to maintain transplant eligibility per the Milan/UCSF criteria [38]. TACE can also be combined with unilateral portal vein embolization (PVE) to induce hypertrophy of the contralateral future liver remnant before hepatectomy of the diseased liver [67]. In patients who have portal vein invasion, chemoembolization plus radiotherapy may be beneficial if hepatic compensation is adequate [68,69]. For advanced-stage patients (BCLC C), TACE may still be useful in combination with the systemic drug sorafenib, but definitive evidence is lacking [70,71]. Thus, TACE provides a versatile tool in the arsenal of treatment options for HCC patients.

Absolute contraindications for TACE include decompensated cirrhosis (Child-Pugh B8 or higher), severely reduced portal vein flow, creatinine clearance <30 mL/min, extensive bilobar tumor involvement, and technical infeasibility [40]. Relative contraindications include high tumor burden, severe comorbidities, untreated esophageal varices, and elevated liver function markers [64]. Generally, lobar and selective/segmental TACE may still be performed with a total bilirubin level up to 3 and 4 mg/dL, respectively [72]. In Child-Pugh class C patients, the American Association for the Study of Liver Disease (AASLD) guidelines recommend against TACE if serum bilirubin is above 3 mg/dL or there is main portal vein thrombosis unless segmental treatment is possible [73,74]. However, Luo et al. noted significant survival improvement in patients with either segmental-branch or first-border branch portal vein thrombosis [75].

### 3.3. Prognostic Factors and Outcomes

As mentioned previously, there are several prognostic models for predicting survival in HCC. The Child-Pugh score may be the most accurate for patients treated with TACE [76]. To determine prognosis for patients undergoing retreatment with TACE (and to decide whether there would be additional benefit after two TACE treatments), the Assessment for Retreatment with TACE (ART) scoring system was developed [8,77].

While the outcomes for both c-TACE and DEB-TACE are more favorable than best supportive care or other conservative management in appropriately selected patients [59,78,79,80,81,82,83], the superiority of c-TACE versus DEB-TACE remains somewhat controversial. At least 12 studies have investigated superiority between the two techniques, but a significant difference in overall survival remains unconfirmed [6,84]. However, the well-known PRECISION V study did demonstrate significant increase in tumor response, reduction in severe hepatotoxicity, and lower doxorubicin-related adverse events in the DEB-TACE group compared with c-TACE for certain patient populations (Child-Pugh B, ECOG 1, bilobar disease, recurrent disease) [81].

The idea of combining locoregional therapy with systemic chemotherapy has been explored. Sorafenib, the first-line treatment for advanced stage (BCLC class C) HCC patients as established by the SHARP trial [85], is both an inhibitor of the growth and proliferation Raf pathway in tumor cells as well as an inhibitor of the angiogenic VEGFR/PDGFR pathway in endothelial cells [86]. The compensatory angiogenesis from TACE-induced hypoxia could theoretically be attenuated from the antiangiogenic functions of sorafenib. Unfortunately, studies like the SPACE trial that randomized patients into DEB-TACE with sorafenib or DEB-TACE with placebo have not shown significant improvements in time to progression [70].

## 4. Transarterial Radioembolization

### 4.1. Procedure and Periprocedural Management

Selective internal radiotherapy (SIRT) for HCC can be performed with transarterial radioembolization (TARE) [10]. This procedure primarily provides its therapeutic effect via radiation instead of embolization [87]. Currently, a radioisotope of yttrium, 90Y, is either loaded onto or embedded within microspheres that are injected into a hepatic artery branch feeding tumor cells [6]. 90Y undergoes beta decay and irradiates surrounding tumor, ultimately damaging repair mechanisms and facilitating cell death [88].

Preprocedural angiographic mapping and evaluation are usually conducted 1–2 weeks prior so that variant anatomy and intrahepatic portosystemic shunts can be identified. Technetium-99m labeled macroaggregated albumin (99mTc-MAA) is used with single-photon emission computed tomography (SPECT) to determine the hepatopulmonary fraction that, if high, may increase the likelihood of radiation pneumonitis after TARE [10,88]. Some patients with advanced hepatobiliary malignancies are prescribed gemcitabine. This chemotherapeutic agent should be held for at least 4 weeks prior to TARE and 2–4 weeks afterwards due to its radiosensitizing effects which increase the risk of radiation-induced liver disease (RILD) [89,90,91]. The actual TARE procedure is performed in similar fashion to other locoregional endovascular approaches with targeting of the tumoral disease in a lobar or segmental fashion [92,93]. Treatment effect is observed slightly later than with TACE or TAE, so follow-up imaging and labs usually take place 12 weeks after TARE [94].

### 4.2. Patient Selection

The indications and contraindications for TARE are generally similar to those for the other embolotherapies. A total bilirubin up to 2 mg/dL is acceptable while encephalopathy and prior radiation to the liver are not [93]. A notable contraindication to TARE is significant (>20%) hepatopulmonary or hepato-enteric shunting as unintended radiation to the lungs or gastrointestinal tract may be serious [6]. However, TARE offers a unique application in patients with portal vein thrombosis given the reduced embolic effect [94,95]. Several series have demonstrated the safety of 90Y-SIRT in cases where tumor infiltrated either a main or lobar portal vein branch [96,97]. Although the BCLC guidelines recommend chemoembolization as first-line therapy for class B patients, expert recommendations from AASLD and NCCN do not posit radioembolization’s inferiority in the list of suitable treatments for unresectable intermediate-stage HCC patients [98,99]. For BCLC 0 and A patients, radiation segmentectomy with intraarterial 90Y-SIRT is safe and effective [100,101]. Neoadjuvant radiation lobectomy is also a safe and effective option to increase the function of the contralateral future liver remnant in patients who plan to undergo resection and avoids the alternative risks of portal vein embolization [102,103]. Finally, just like the other embolotherapies, TARE can be used to maintain or encourage transplant/resection eligibility through bridging as well as enhance overall survival in BCLC C patients [104,105].

### 4.3. Prognostic Factors and Outcomes

Prognosis after TARE is most associated with baseline patient stage (BCLC, Child-Pugh), performance status (ECOG), tumor burden, and extrahepatic disease [10]. According to a 2016 meta-analysis by Lobo et al. [106], overall survival and complication rates for TARE are similar to those of TACE, but the prospective trial PREMIERE demonstrated longer time to progression (TTP) for TARE [107]. Another randomized trial showed higher quality of life scores for TARE patients vs. TACE [108]. In a prospective study, Salem et al. reported excellent outcomes including an overall survival of 47.3 months for Child-Pugh A patients and 27 months in Child-Pugh B patients [109]. With more contemporary approaches such as radiation segmentectomy, response rates, tumor control, and survival outcomes have been comparable to curative-intent treatments (e.g., resection, transplantation, ablation) at 5 years [109,110].

When compared to sorafenib among advanced-stage patients, Hilgard et al. [111] actually demonstrated a survival benefit for TARE (10.7 vs. 16.4 months, respectively). Two randomized controlled trials revealed higher tumor response rates and fewer adverse events with TARE vs. sorafenib for unresectable, treatment-naïve Child-Pugh A patients, although overall survival was similar between the two [112,113]. Considering the side effects of systemic sorafenib therapy, TARE may be an attractive option for these patients [113].

## 5. Ablation

### 5.1. Procedure and Periprocedural Management

Ablative techniques for HCC include radiofrequency ablation (RFA), microwave ablation (MWA), cryoablation (CA), irreversible electroporation (IRE), laser-induced interstitial thermotherapy (LITT), and high-intensity focused ultrasound (HIFU). The choice of technique is often based on individual and institutional expertise, but historically the most commonly utilized technology was RFA. In recent years, MWA has gained traction as an alternative modality [114]. CA has also been used regularly in the past, although its use has diminished due to serious complications including cryogenic shock, acute renal failure secondary to myohemoglobinuria, coagulopathy, and cardiac dysrhythmias [115,116].

In RFA, the tumor is heated to high temperatures via frictional heat in water molecules produced by an electrode, which can be with or without hooks, to increase and maximize heat production in the tissue [117]. The ablation zone consists of the original space occupied by the tumor plus a 5-10 mm boundary of ablated adjacent liver parenchyma [118]. Over time, fibrosis causes retraction of the necrotic tissue. A homogenous, non-enhancing, well-circumscribed area is consistent with successful radiographic response [118,119]. Conversely, MWA uses an electrode to deliver thermal energy-induced cellular destruction. MWA may be suitable for larger tumors than RFA is indicated for and highly perfused regions [120]. Moreover, MWA can target multiple tumor sites simultaneously, yields shorter time to threshold temperature, achieves larger ablation treatment zones, results in better delineated ablation zone borders, and is less prone to heat-sink effects from adjacent vascular structures [114,120,121]. While RFA yields smaller ablation zones, less uniform borders, and is more prone to heat-sink, it does offer the advantage of avoiding energy delivery to tracking structures such as bile ducts or large vessels [122].

All ablative modalities utilize imaging guidance/monitoring during the procedures such as computed tomography (CT) or ultrasound (US), or a combination. New modalities such as contrast-enhanced US (CEUS) have also been utilized as well as novel fused imaging technologies to predict treatment zones [123,124]. Follow-up imaging with CT or MRI every 3–6 months for the first two years with serum alpha fetoprotein (AFP) monitoring is recommended by the NCCN [99]. Serious complications include injury to adjacent organs e.g., diaphragm, gastrointestinal tract, gallbladder [125]. Similar to PES, a postablation syndrome (PAS) may occur with analogous clinical presentation and self-limiting nature [126].

### 5.2. Patient Selection

Surgical resection or orthotopic liver transplantation is the mainstay of treatment for very early and early-stage (BCLC 0, A) HCC patients [127]. However, most patients are disqualified from surgical intervention due to significant comorbidities, portal hypertension, poor hepatic function, cardiovascular comorbidities, inability to tolerate general anesthesia, or lesion location [5,128]. As such, ablation offers a potentially curative option for these patients with early HCC with some studies demonstrating equivalent survival outcomes to resection even with poorer baseline liver function [129,130,131]. Ablation with curative intent is an effective alternative to resection, particularly for tumors smaller than 3 cm [114]. For tumors 3–5 cm in diameter, the combination of TACE and ablation demonstrate good outcomes albeit not curative [132,133,134,135]. Caution must be exercised when lesions are close to major vessels, biliary structures, diaphragm, and other intra-abdominal organs. Hydrodissection, or artificial ascites, wherein 5% dextrose in water (D5W) fluid is injected between the tumor area and adjacent extrahepatic organ to prevent transmission of thermal energy, helps to separate the tumor and protects against unintentional organ injury [122,136,137]. RFA and MWA can also be considered in advanced stage patients (BCLC C) for downstaging as a bridge to transplantation and in intermediate stage patients (BCLC B) when combined with TACE [98].

### 5.3. Prognostic Factors and Outcomes

The independent predictors of survival after RFA/MWA from several multivariate analyses were Child-Pugh class, tumor size, and tumor number [138,139,140,141,142]. The overall survival outcomes between RFA and resection are not significantly different at 1 and 3 years [143,144,145], but MWA may see lower local tumor progression rates than RFA for tumors > 5 cm or > 3 HCC nodules [120]. Complication rates are similar between MWA and RFA [146]. Although there is a relative paucity of high-impact studies pertaining to cryoablation, an RCT by Wang et al. found higher 3-year survival rates (but similar 5-year survival rates) and lower local progression rates (for tumors > 3 cm) in the cryoablation group vs. RFA group [147].

Combination therapy of RFA with chemoembolization has been investigated and has shown improved locoregional control compared to either RFA or TACE alone for BCLC A and B patients [148]. RFA helps to decrease total cellular resistance so that chemotherapy in TACE can yield relatively higher concentrations proximal to the tumor vascular bed at the periphery of the ablated tissue [149]. If TACE is conducted first, peripherally situated tumor cells are preferentially destroyed so that later RFA treatment, typically performed within 4 weeks, undergoes less vascular heat sinking and yields more complete central necrosis particularly for lesions > 3 cm [150]. The optimum parameters for both mechanisms require further exploration. MWA plus TACE has also shown to be effective for lesions between 3–5 cm [133,134,135]. It should be noted that adjuvant sorafenib therapy following ablation or resection is not effective per results from the STORM trial [151].

## 6. Future Directions

New developments in drug-eluting bead technology have allowed the loading of tyrosine kinase inhibitors (e.g., sunitinib, vandetanib) and anti-VEGF antibodies (e.g., bevacizumab) in preclinical stages with promising results in halting tumor growth [152,153,154,155]. Experiments in immunotherapy such as oncolytic viruses, dendritic cells, and immune checkpoint inhibitors (against CTLA-4, PD-1, PD-L1) are also underway [156,157]. In fact, nivolumab, a PD-1 inhibitor (already FDA approved for sorafenib-refractory HCC) is being directly compared to sorafenib for advanced stage HCC patients [158,159]. Several combination strategies of innate and adaptive immunotherapies with RFA, MWA, and cryoablation are also be investigated in vitro and in animal models of HCC [160]. Lastly, personalized therapies and prognosticators are being appraised in human subjects research. Several putative histological, epigenetic, and metabolomic biomarkers are being studied to individualize treatments for HCC patients [161]. For example, micro-RNA-122 (miRNA-122) is a tumor suppressor molecule that is often severely reduced in hepatocytes linked to hepatocellular oncogenesis. Therapies that involve reintroduction of miRNA-122 to stabilize cell cycle regulation are being scrutinized for effectiveness and safety [162,163]. Advances in artificial intelligence (AI) are also being applied to HCC management. In addition to improved intra-procedural imaging guidance, AI has been used to construct prediction models for response to locoregional treatment [164,165]. While the role of interventional-based liver-directed techniques continues to expand, additional research is needed regarding the application of these therapies in a neoadjuvant or adjuvant setting to improve the multidisciplinary care of HCC and reduce recurrence rates [166].

## 7. Conclusions

HCC is the most common primary liver malignancy and the third leading cause of cancer-related mortality worldwide [1]. Although overall survival for this complex disease has improved in recent years, prognosis is still poor particularly for advanced and terminal-stage patients [1]. Surgical extirpation and transplantation remain the curative standard of care for early-stage patients, but there is an expanding role of locoregional therapies in the management of HCC including curative-intent, disease control, bridging to transplant and resection, downstaging patients, and palliation. With the addition of targeted chemotherapy and radiotherapy delivery, the inventory of transarterial hepatic embolization techniques offers major benefit in appropriately selected candidates. Ablative procedures using high frequency alternating currents or microwaves have also developed as excellent therapies for nonsurgical patients, achieving curative results in early-stage patients. Although advanced-stage patients are currently limited to systemic therapy, novel advances in immunotherapy and personalized biomolecular signatures of HCC are paving the way for more robust strategies to tackle this disease.

## Figures and Tables

**Table 1:** Summary of Locoregional Therapy Options for Hepatocellular Carcinoma.

Modality	Techniques	Clinical Utility	Risks	Benefits
TAE	Particulate or liquid embolic agents	Disease control (BCLC B) and bridging/downstaging to transplant (BCLC A, B).	PES, liver failure, liver abscess/biloma	Improves OS vs. best supportive care. Avoids chemotherapy toxicity. Less expensive than TACE.
TACE	Conventional emulsified chemotherapeutic agent (c-TACE) or drug-eluting beads (DEB-TACE)	Same as TAE. Can combine with portal vein embolization before resection.	PES, liver failure, liver abscess/biloma	Improves OS vs. best supportive care. Simultaneous embolic and chemotherapeutic effects.
TARE	Yttrium-90 radioisotope loaded onto microspheres	Same as TAE/TACE. RS for nonsurgical early stage patients (BCLC 0, A). Can also be used in portal vein thrombosis.	RILD, radiation-induced pneumonitis, PES, liver failure, liver abscess/biloma	Higher quality of life/TTP vs. TACE. RS outcomes comparable to curative-intent treatments (e.g., resection and ablation) at 5 years
Ablation	Microwaves, radiofrequency alternating current, laser, cooling	Early stage HCC < 2–3 cm in non-surgical candidates (BCLC 0, A). Improved outcomes for tumors 3–5 cm when combined with TACE.	PAS, bleeding, adjacent organ injury	Similar outcomes as resection for tumors < 3 cm.

PES—postembolization syndrome. PAS—postablation syndrome. OS—overall survival. RILD—radiation-induced liver disease. CP—Childs-Pugh class. RS—radiation segmentectomy. TTP—time to progression.
